# Language-Based Access to Large Sensor Repositories

**DOI:** 10.3390/s90402926

**Published:** 2009-04-22

**Authors:** Peter Baumann

**Affiliations:** Jacobs University Bremen, Campus Ring 12, 28759 Bremen, Germany; E-Mail: p.baumann@jacobs-university.de; Tel. +49-421-200-3178; Fax: +49-421-200-493178

**Keywords:** Sensor data management, raster services, standards, OGC, WCPS, WCS

## Abstract

Sensor data have broadened their scope recently, ranging now from the simple time series measurements to, e.g., hyperspectral satellite image maps timeseries. In addition to observed data, simulation data increasingly have to be merged, for example 4-D ocean and atmospheric data. The majority of these data fall into the category of multi-dimensional rasters. However, when it comes to flexible retrieval, including sensor data search, aggregation, analysis, fusion, etc., standard query language support in the past has not kept up with the service level of, e.g., metadata retrieval. To close this gap, the Open GeoSpatial Consortium (OGC) has issued the Web Coverage Processing Service (WCPS) Standard in December 2008. WCPS defines a request language for multi-dimensional raster data, suitable for specifying navigation, download, and analysis of sensor, image, and statistics data. This contribution emphasises sensor data modeling and the perspectives for an integrated, cross-dimensional sensor data retrieval. Further, the WCPS reference implementation is briefly discussed.

## 1. Motivation

Sensor data contribute substantially to today's geo data mix. An ever-increasing number of instruments with a plethora of individual characteristics deliver data which needs to be received, actively polled, homogenized, stored, evaluated, and fed forward to human users for inspection and decision making or, via automated chaining, to tools for further analysis.

Technically, measurements can often be represented as raster data of some particular dimension, such as 1-D timeseries, 2-D imagery, 3-D image time series or geophysical data, 4-D climate/ocean data, and n-D statistics data with “abstract”, non-spatiotemporal axes. While today's efforts still emphasize mere data availability through open, easy-to-navigate extraction interfaces, the upcoming trend of “Data as a Service” (DaaS) suggests transforming data stewardship into service stewardship with flexible, on-demand analysis capabilities.

Use of open standards seems indispensable in view of the large, disparate communities to be served, and also their increasing demands (or pressure, depending on the viewpoint) for integration. In the family of open geo standards developed and maintained by the Open GeoSpatial Consortium (OGC, www.opengeospatial.org) it is the Sensor Web Enablement (SWE) suite of standards which provides interface specifications for open access to heterogeneous sensor networks [1]. Like other OGC standards, it too relies on the Geography Markup Language (GML) [2] and the compulsory baseline definitions of OWS Common [3]. For raster data access, OGC offers the Web Coverage Service (WCS) standard which provides open, interoperable raster (ie, “coverage”) data access [4]. WCS defines a service interface for data extraction based on spatial and temporal subsetting, range (“band”, “channel”) subsetting, scaling, reprojection, and data format encoding.

This suite of standards helps to access data, but it does not allow versatile retrieval and processing with a quality similar to what, e.g., SQL accomplishes on alphanumeric data. Therefore, since December 2008 the tentatively simple raster data subsetting of WCS is complemented by the Web Coverage Processing Service (WCPS) standard, which adds a coverage processing language for flexible *ad-hoc* navigation, extraction, and analysis of multi-dimensional raster data [5]. Sensor, image, and statistics data offered by some server can be addressed through requests of unlimited complexity, due to the language approach; for this reason WCPS has been dubbed “SQL for coverages”.

Core goals in the design of WCPS have been to combine expressiveness, flexibility, usability, optimizability, and safety in Web environments. Expressiveness aims at allowing a large range of sensor, imaging, and statistics functionality, including cross-dimensional and cross-domain operations. Flexibility is needed because a set of predefined functions will never be able to accommodate current and future needs in the manifold application domains anticipated; a language-based approach seems to be the only viable way. Usability addresses the understandability of the specification document; while a mathematically formalized semantics serves best for a clear, unambiguous conceptualization, it cannot be assumed that all implementers are familiar with such techniques; therefore, a semi-formal approach was adopted. Only a sufficiently high-level, declarative language will be optimizable, i.e., leave room for the server to rephrase incoming requests for best execution performance. The database domain has a rich body of experience there, so this was duly considered. Good practice in databases is also to design query languages “safe in evaluation”, which means that no single request can block a server for unlimited time. For a detailed discussion of the design rationales see [6].

At the moment front-end services offering access to consolidated sensor data repositories certainly constitute a core application domain of WCPS. However, WCPS is also useful for upstream sensor data access whenever non-trivial on-the-fly filtering and data processing is required.

In this contribution we present WCPS with emphasis on sensor retrieval tasks. Findings presented stem from our active work in OGC, which includes advancing the WCS specification as co-chair of the respective working group, development of the WCPS specification, and architecting its reference implementation.

The remainder of this contribution is organized as follows. In the next section, the main concepts of the WCS coverage model, the WCPS language, concrete protocol embeddings, and the reference implementation service stack are presented. Section 3 illustrates application of WCPS by means of scenarios covering 1-D to 4-D sensor data. Section 4 concludes the paper.

## 2. WCPS

In this section, we first introduce the notion of a coverage. Then, core WCPS language constructs are introduced and exemplified. Finally, a brief discussion of the protocol embeddings and the reference implementation is given.

### 2.1. The WCS Coverage Model

ISO 19123 [7] and OGC Abstract Topic 6 [8], which technically are identical as they are mutually adopted by ISO and OGC, normatively define the term *coverage* as being a “feature that acts as a function to return values from its range for any direct position within its spatial, temporal, or spatiotemporal domain”. Coverage types are grouped into discrete and continuous, both of which are subdivided further into various regular and irregular variants. Based on this abstract notion, The Web Coverage Service (WCS) standard defines a concrete coverage data structure (Additionally, GML contains a built-in model for small-scale coverages; as this is suitable only for special cases of raster data, hence we disregard it here.) for the discrete point coverage subtype – i.e., raster data –and an access service based on this notion.

A coverage basically is a function which maps coordinate locations to values. It is materialized as a multi-dimensional value array, containing cells (“pixels”, “voxels”) at the grid locations. The set of admissible coordinate values is called the coverage's domain, which is spanned by a number of axes (or dimensions) defining the coverage's dimensionality. For each axis, the coverage is delimited by some lower and upper bound, expressed in some coordinate reference system (CRS). Each coverage has a list of CRSs associated in which it can be queried; requesting values in another CRS than the one in which the coverage is stored (or in the image coordinate system, directly using pixel coordinates) obviously will involve reprojection.

A coverage array can be of one, two, three, or four dimensions, comprised of x, y, z, and time axes. Coverages are allowed to have any combination of axes, including, for example, 1-D time-only sensor time series, 2-D x/z planes, or 5-D x/y/z/time/pressure cubes. For the future it is foreseen to additionally allow so-called abstract axes with application-defined semantics (such as products offered by a company).

WCPS slightly extends this notion by adding specific axis semantics. Axis types provided are *x, y, z* for Cartesian coordinates, *r* and *phi* for polar coordinates, and *t* for time. In future, additional user-defined axes without spatio-temporal semantics will be supported, such as pressure.

The structure of a coverage's cell values (denoting the set of all possible values associated with a cell) is given by its range type. Range values can be atomic, or a list of named components called range fields (commonly known as “bands” or “channels”). Range fields, in turn, can be atomic or can consist of multi-dimensional arrays of values themselves (The latter feature is recognized as being relatively complex to implement and handle; hence, it is optional now and is likely to be factored out into a bespoke extension in the next WCS version). With each range component a set of possible interpolation method can be associated, one of which can become default; they are specific to each component because interpolation (like summarizability) depends on the actual semantics of data: visual images can be interpolated, while land use data cannot. Interpolation methods available are *none*, *linear*, *quadratic*, and *cubic*, with the obvious meanings. Further, the concept of null resistance serves to control the impact of null values when interpolating over a set of cells of which at least one has a null value.

In WCPS, range types actually are less general than in WCS, to allow for well-defined processing semantics: only the numeric data types known from programming language are available, plus complex numbers in single and double precision, as well as single-nested records. This allows expressing SCADA sensors, hyperspectral satellite imagery, elevation and bathymetry data, and spectral data, to name but a few.

WCS provides three request types on such coverages when offered by a server. Following OGC convention, the first is *GetCapabilities*. This request informs a client about the procedural capabilities it has (such as the data formats supported) and gives a list of the coverages offered. Detailed metadata about each coverage can be obtained via the second request type, *DescribeCoverage*. The workhorse, finally, is the *GetCoverage* request type which serves to extract data from some coverage by applying spatiotemporal subsetting, range component (“band”) subsetting, reprojection, scaling, and format encoding of the result. An interpolation method can be indicated in the request to enforce a method other than the default when rescaling or reprojecting.

Care has been taken in the design of WCS that it is always possible to request a coverage (or a subset of it) with cell values untouched, retaining their original values. For example, while there may be many CRSs in which a given coverage can be accessed it is required that there is always one particular, so-called Image CRS which allows to retrieve data in cell (i.e., integer) coordinates. On a side note, this is a main difference to the Web Map Service (WMS) standard which serves to portray features and coverages as images aimed at human consumption only.

### 2.2. Coverage Processing Language

Below we introduce the WCPS language. Following a presentation of the overall request structure we illustrate representative processing functions.

#### 2.2.1. Overall Structure

The overall structure of a WCPS request, which is related to XQuery, follows this syntax scheme:

for $*var_1_* in (*cov_1,1_, cov_1,2_*, …),

    …

    
$*var_n_* in (*cov_n,1_, cov_n,2_*, …)

where

     
*filter_predicate*(*$var_1_*, …, *$var_n_*)

return

     
*processing_expression*(*$var_1_*, …, *$var_n_*)

As the syntax suggests, this denotes an n-fold nested loop where in each loop a variable successively is bound to the coverages listed. In each iteration, the filter predicate is applied first; only if it evaluates to *true* will the processing expression be evaluated so that it can contribute a result item. The outcome of such a request is a list of either coverages or, for scalar-valued processing expressions, a list of scalars. A processing expression mainly consists of calling the *encode*() function which prepares a coverage-valued result for shipping to the client using a suitable data format.

*Example:* “Coverages *WaterTemperature* and *AirTemperature*, each encoded in CSV (comma-separated values).”

for $t in (WaterTemperature, AirTemperature)

return

    
encode($t, “csv”)

By applying the *store*() function, the result optionally is not shipped directly, but stored in the server for subsequent retrieval through the client which, for this purpose, gets a URL for each result coverage instead of the coverage itself. In this case, the previous example can be rewritten as

for $t in (WaterTemperature, AirTemperature)

return

    
store(encode($t, “csv”))

Core to WCPS is the coverage expression where, based on the variables to which the stored coverages are bound, a new, transient coverage is derived. This will be inspected next.

#### 2.2.2. Value-Manipulating Functions

To modify values in a coverage, induced operations can be used. This concept, which traces back to Ritter *et al.* [9], allows to apply any operation that is available on the cell type to all cells of a coverage simultaneously, regardless of its dimensionality.

*Example:* “The logarithm of the river water temperature, transformed from Fahrenheit to Celsius.”

for $r in (RiverSensor)

return

    
encode(log(9 / 5 * $r.temp – 32), “csv”)

Induced operations, which can be unary and binary, are provided for Boolean, arithmetic, trigonometric, and exponential operations. Further, coverages containing complex records (“structs”) as cell values can be constructed this way. A cast operator allows to adjust data types and lengths.

#### 2.2.3. Domain-Manipulating Functions

So far we have manipulated the cells of a coverage, but not its extent. This is done with subsetting operations. The *trim*() operation cuts out a sub-coverage by way of a given bounding box. The dimension of the coverage remains unchanged. This is different with the *slice*() operation where, at the specified position in the original coverage, a lower-dimensional cutout is produced. For trim and slice operations there is a convenient shorthand notation using brackets. For reasons of completeness there is also an *extend*() operation which grows a coverage while maintaining its dimensionality; the new positions are filled with null values.

Along with the subsetting operations, coordinates come into play. These can be expressed in some coordinate reference system (CRS) supported by the coverage on hand or simply in the coverage’s integer cell coordinates. Axis types predefined with the standard are *x* and *y* for cartesian coordinates, *r* and *phi* for polar coordinates, *z* for elevation (i.e., height or depth), and *t* for time. Conceptually, WCPS is prepared for further axis with a non-spatiotemporal semantics, so-called *abstract* axes, once WCS supports these.

Let us first inspect direct access using cell coordinates.

*Example:* “From my MODIS image, the first 100 by 100 pixel.”

for $m in (ModisImage)

return

    
encode($m[x(0:99), y(0:99)], “png”)

As can be seen, the lower and upper bound is indicated for each dimension individually and identified by the axis name. Therefore, the sequence of axes within the brackets can be arbitrary. This we next extend by indicating a CRSs in which the coordinates provided are expressed. For each CRS supported by OGC a URN is assumed to be available, to be maintained by the forthcoming OGC naming authority. For example, the well-known EPSG list of CRSs is already defined. For a coverage available in the WGS84 system a trim operation might be as follows.

*Example:* “From my MODIS image, a cutout between corner points (−10,40) and (+20,60), expressed in WGS84.”

for $m in (ModisImage)

return

    
encode(

      
$m[x:“ogc:urn:def:crs:EPSG::4326”(−10 : 20),

      
y:“ogc:urn:def:crs:EPSG::4326”(40 : 60)

       
],

      
“hdf-eos”

    
)

The only time coordinate system supported currently is ISO 8601 [10]. Time coordinates can be applied just like with the geo coordinates before.

*Example:* “From time series *WaterTemperature*, all values between February 1, 2009 and February 20, 2009.”

for $t in (WaterTemperature)

return

    
encode(

     
$t[t:“ogc:urn:def:crs:ISO::8601”

        
(“P2009Y02M02D” : “P2009Y02M20D”)

      
],

     
“csv”

    
)

Another extent changing function is scaling. Again, axes are treated independently from each other so that any combination is possible. This is particularly relevant when it comes to non-horizontal scaling where the scale factors are not coupled like it usually is the case with the *x* and *y* axis. However, scaling needs an additional parameter set which is due to the inherent complexity of this operation. As in WCS, the scaling operation allows to specify which interpolation method to apply in case that resampling occurs. However, we omit discussion of this mechanism and refer to the WCS specification [4] for further details.

*Example:* “All salinity measurement values, scaled down to a list of size 100, using default interpolation.”

for $s in (SalinityColumn)

return

    
encode(scale($s, {z(0:99)), “csv”)

Yet another complex operation, both conceptually and in evaluation, is a reprojection. Function *crsTransform*() receives a coverage-valued argument plus, for each axis, the target CRS. As with scaling, the interpolation method to be applied can be specified.

*Example:* “My MODIS image, reprojected to EPSG:63266405 using default interpolation.”

for $m in (ModisImage)

return

    
encode(

      
crsTransform(

       
$m,

       
{x:“ogc:urn:def:crs:EPSG::63266405”,

        
y:“ogc:urn:def:crs:EPSG::63266405”

       
},

       
{}

      
),

      
“jpeg”

    
)

The empty set provided in the above expression specifies default itnerpolation; alternatively, specific interpolation methods can be invoked per range component. The above functions allow performing a substantial part of the day-to-day sensor analysis and retrieval tasks. We now inspect some of the advanced functionality.

#### 2.2.4. Aggregation

Aside from returning selected and processed coverages, it is often required to obtain summary data about one or more coverages. To this end, WCPS provides aggregation operations similar to SQL, but extending it with coverage-specific operators. Well-known from SQL are aggregation functions like *count*, *average*, *max*, *min*, and the existential quantifiers *all* and *exist*. All of them are available on coverage cells as well, with the intuitively expected semantics.

*Example:* “The maximum temperature measured by sensor *WaterTemperature*.”

for $w in (WaterTemperature)

return

    
max($w)

No coverage encoding is performed here as the result is a scalar. Such values are transferred as ASCII numbers; in future, XML encodings may be considered in addition. A prominent place where these so-called condensers (which have been named like this to differentiate language wise cell array from set processing) appear is the filter predicate. Here condensers serve to express statements about coverages which need to be fulfilled to allow them participating in the result list.

*Example:* “All sensors where, sometime in 2008, threshold *T* was exceeded.”

for $s in (Sensor1, Sensor2, …)

where

    
some(

      
$s[t:“ogc:urn:def:crs:ISO::8601”(“P2008”:“P2009”)

        
] > T

    
)

return

    
identifier($s)

These so-called condensers actually represent specializations of a more general construct. A general condenser inspects all cells of a given extent, applies some evaluation function on them, and aggregates the result using the summation function specified. The syntax is as follows:

condense  *summation*

over    *name_1 axis_1* (*extent_1*),

      
…,

      
*name_n axis_n* (*extent_n*)

using  *value_expression* (*name_1, …, name_n*)

For summation, operators available are *+*, *, *max*, *min*, *and*, and *or*. The *name_i* variables iterate over the extent of their respective axis. For each position of the new coverage, the value expression – which may contain occurrences of the coordinate iterators – is evaluated.

*Example:* “The sum of the absolute changes between subsequent temperature measurements.”

for $w in (WaterTemperature)

return

    
condense   +

    
over     pt t (…)

    
using     abs($w[t(pt)] - $w[t(pt-1)])

#### 2.2.5. Coverage Construction

The coverage constructor creates a new coverage by filling values which are computed on the fly into some extent indicated. The main difference to the induced operations is that the coverage constructor provides explicit coordinates which can be used in the computation of new values. The syntax is:

coverage  *fieldname*

over      *name_1 axis_1* (*extent_1*),

        
…,

        
*name_n axis_n* (*extent_n*)

values    *value_expression*(*name_1, …, name_n*)

The field name is the name which the single range field component will receive; while not necessary for a single-component coverage, it is required by WCS and hence mandatory in the expression. The *name_i* variables iterate over the extent of their respective axis. For each position of the new coverage, the value expression – which may contain occurrences of the coordinate iterators – is evaluated.

Actually, induced operators are a special case of the coverage constructor. Assume a coverage with a single band *b* and a 2-D *x/y* extent is bound to variable $*c*. Then, the induced expression 2*$*c* can be rephrased as:

coverage b

over    px x(…),

      
py y(…)

values     2 * $c[x(px), y(py)]

The careful reader will notice that the result of slicing *$c* is a zero-dimensional coverage. By definition we consider such a single-cell array as equivalent to a scalar. Histograms form a typical application example for the coverage constructor, as the following example shows.

*Example:* “A histogram of the (8-bit) red channel of my MODIS image.”

for $m in (ModisImage)

return

    
encode(

      
coverage histogram

       
over     bucket x (0:255)

       
values      count($m.red = bucket),

      
“csv”

    
)

For each of the 8-bit bucket values the induced comparison of the bucket value is performed against the complete image matrix. The resulting Boolean matrix is passed to the *count* aggregate which determines the number of *true* values, thereby finding the frequency of the bucket under consideration. The axis type – here randomly chosen: *x* – is of no further concern for the result.

This way, completely new structures can be derived from existing coverages. Queries up to the complexity of the Fast Fourier Transform have been expressed with such constructs during the assessment of WCPS prior to its standardization.

#### 2.2.6. Further Features

Cross-dimensional sensor fusion can be expressed by nested loops.

*Example:* “For location x0/y0, the difference between temperature measured in situ and by an AVHRR SST (sea surface temperature) satellite image time series.”

for $i in (InSitu),

    
$a in (AvhrrTimeSeries)

return

    
encode($i - $a[x(x0), y(y0)], “csv”)

Aside from the coverage processing functions introduced so far there is also a set of functions for retrieving and setting metadata of coverages excerpted. To avoid misunderstandings, these are not general or application metadata, but only those ones defined with WCS coverages. Sometimes these are termed “technical metadata” as they are the bare minimum required to correctly interpret the cell data.

Coverage, cell indexing, and arithmetic expressions can be nested arbitrarily, thereby allowing for requests of unlimited complexity. Well-known features from programming languages, like the expected operator precedence, parentheses, and dynamic type adjustment have been incorporated to help immersing into the language quickly.

### 2.3. Protocol Embedding

Tentatively the WCPS language has been defined in a service-neutral manner. The core WCPS specification [5] contains only the language syntax and semantics. Although WCPS has emerged from the work on WCS, it is formulated in a high-level manner and independent from particular OGC service protocols. Actually, there are already two such embeddings already. The first one is defining WCPS as an extension to WCS whereby an additional request type, *ProcessCoverages*, is introduced. In the corresponding standards document [11] concrete service bindings are indicated for HTTP GET and XML/POST. A service implementer can choose to implement only the mandatory, comparatively simple GetCoverage request or additionally the optional, more complex ProcessCoverages request.

The second embedding relates WCPS to the Web Processing Service (WPS) [12]. WPS defines an XML-RPC based interface to geo services where the operational semantics is only given by function name and function parameters. Consequently, WPS implementations are not interoperable per se; the concept rather is that concrete, focused application profiles (in OGC terminology) be defined in an interoperable manner. WCPS is such an interoperable WPS application profile, defined in [13]. In the WPS context, the advantage of the language approach is that not only static functionality can be invoked remotely, but dynamic run-time request composition by clients becomes feasible. Additionally, the well-defined semantics of WCPS expressions opens up avenues for automatic orchestration of service clouds, but also server-internal optimization.

A third connection, to the Sensor Web Enablement (SWE), is under discussion. Hence, WCPS with its abstract concept of “coverage processing as a service” bridges those OGC standards where coverages are relevant. On a side note, this shows how a clear modularization can require splitting of a concept over several specification documents. While sometimes implementers complain about too many specifications, OGC itself works hard on minimizing the amount of standards documents.

### 2.4. Reference Implementation

**Figure 1.**
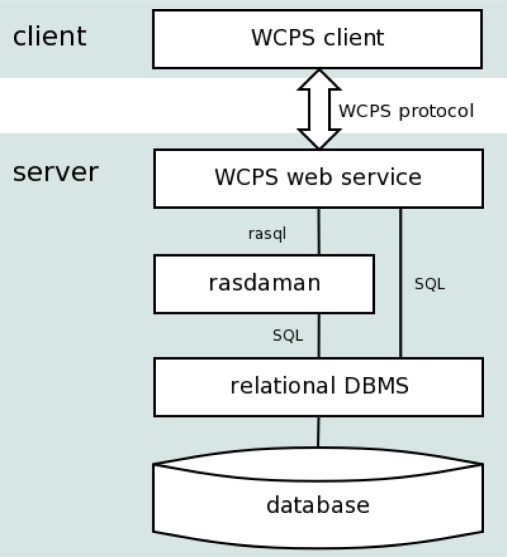
WCPS reference implementation service stack.

The reference implementation of WCPS is based on the rasdaman raster DBMS [1,5,14,15] and the PostgreSQL relational DBMS. Figure 1 shows the overall architecture. WCPS requests are translated into queries of the rasdaman query language, rasql. In the rasdaman server, queries are optimized and then executed against raster objects stored in a standard relational database, partitioned (“tiled”) into Binary Large Objects (BLOBs). Due to the formal semantics of rasql [1,14] there is a stable interface which allows implementations to perform manifold optimizations, including exploitation of hardware parallelism.

Query optimization is a wide research area and one of our core research domains. The following is a list of some important methods currently available:

- Algebraic optimization rewrites a query Q into another query Q' such that Q and Q' deliver the same result, but Q' does so faster. Currently, about 110 optimizing rules are incorporated in the rasdaman rule base. For example, for a given query:
for $c1 in (MyFirstCoverage),
      
$c2 in (MySecondCoverage)
return
      
sum($c1 + $c2)
  the rule “*sum*(*a* + *b*) ≡ *sum*(*a*) + *sum*(*b*)” allows to substitute the above query by the one below, effectively changing the induced operation “+” (which requires one addition per cell) into a scalar addition (which requires only one addition at all) and, thereby, saving 1/3 of the main memory processing cost.
for $c1 in (MyFirstCoverage),
      
$c2 in (MySecondCoverage)
return
      
sum($c1) + sum($c2)
- Parallel evaluation allows both concurrent database access and performance increases by tasking more than one CPU with answering a query. Concurrent processes can run on the same server or in a cluster.
- Just-in-time compilation determines suitable query fragments, generates C code ready to process one tile in the way specified, compiles this into a shared library, loads this into the server executable, and executes it [16]. Once such a library has been loaded, it can be reused with subsequent queries of same structure, but possibly varying parameter values. Preliminary performance evaluations show that query evaluation gets close to the speed of hand-crafted code.
- Alternatively, the query fragments found can be mapped to Graphic Processing Unit (GPU) code for processing in parallel to the CPU, which can evaluate the remaining part of the query meantime [17]. The gain in parallelism is substantial due to the many cores a GPU provides.

## 3. Use Case Scenarios

Following the functionality-driven presentation of the WCPS language, we now focus on some real sensor data retrieval use cases available on the Web. The www.earthlook.org demonstration website has been developed to showcase WCPS retrieval on 1-D to 4-D base data sets. The following subsections discuss these. At www.earthlook.de/tech/wcps-tutorial/sandbox-abs.php there is also a “sandbox” available which allows typing in and submitting own requests against sample data sets.

### 3.1. One-Dimensional Data

#### 3.1.1. Scenario 1

**Figure 2.**
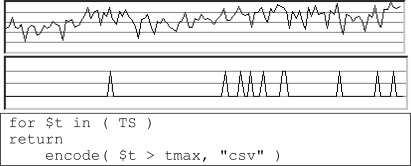
Sample time series (top) and threshold indicator array (bottom).

Sensor *TS* delivers scalar values (such as temperature values). Task on hand is to determine times when threshold *tmax* has been exceeded. The result of this request is a 1-D array containing *true* for values above and *false* for values below threshold, encoded as comma-separated values (see Figure 2).

#### 3.1.2. Scenario 2

The above sensor now needs to be monitored continuously. To this end, a simple watchdog process periodically requests the current value. Nothing is done as long as the result is *false*, but some action (like sending an e-mail or SMS) is performed for a *true* result. The underlying query, which returns such a Boolean result value, is:

for $t in (TS)

return

    
$t[

      
t:“ogc:urn:def:crs:ISO::8601”(“P2009Y02M22DT16H13M24S”)

     
] > tmax

On EarthLook, two different scenarios are provided: fetching the current value and fetching the average over all values seen so far.

### 3.2. Two-Dimensional Data

**Figure 3.**
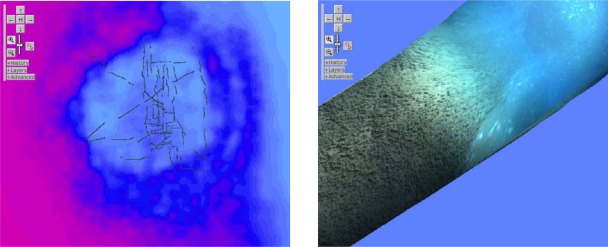
Overview of the *Håkon-Mosby Seafloor Mud Volcano* (left) and detail zoom (right), obtained as EarthLook screenshots (data acquired on AWI Polarstern cruise *ArcXIX3b* [13]).

The core application of 2-D data obviously is mapping. The standard use case here is a map overlay presented for zoom and pan, as the OGC Web Map Service (WMS) specifies. From the examples provided on EarthLook, we discuss the more complex one. For the area of the Northernmost known underwater mud volcano, *Håkon-Mosby,* four map layers are provided: one bathymetry layer and three video mosaics obtained from a camera on board a Remotely Operated Vehicle (ROV) of type Ifremer Victor6000 [18]. These have been ingested into a rasdaman database (see Section 2.2.4) where they are available through a WMS interface. Figure 3 shows screenshots made using the WMS client coming with rasdaman. In the top left part of each screenshot the navigation controls are visible. In this use case we inspect the structure of the queries submitted by the client.

#### 3.2.1. Scenario 4

The client fetches images piecemeal. Each individual query is a WMS request which can be emulated by the following WCPS request. We assume bounding box coordinates (*x_0_,y_0_*) / (*x_1_,y_1_*) expressed in the coverage’s native CRS, say, UTM Zone 33N.

for $b in (HM-Bathymetry),

    
$d1 in (HM-Dive1),

    
$d2 in (HM-Dive2),

    
$d3 in (HM-Dive3)

return

    
encode(

      
scale(

        
(

          
(

            
($b < −1300)

              
* struct{red:0, green:0, blue:0}

        
+  (−1300.000000<$b and $b <= −1290)

              
* struct{red:219, green:0, blue:172}

                    
*…28 more summands like this…*

        
+  (−126.5 < $b)

              
* struct{red:255, green:255, blue:255}

        
)

        
overlay $d1

        
overlay $d2

        
overlay $d3

        
)  [x:“urn:ogc:def:crs:EPSG::4326”(x_0_:x_1_),

            
y:“urn:ogc:def:crs:EPSG::4326”(y_0_:y_1_)

        
],

        
{ x(0:299), y(0:299)},

        
{ }

      
),

      
“jpeg”

    
)

The highest processing load comes from the *ad-hoc* depth classification into 31 colors altogether. Every line contains a predicate which filters out a particular depth interval. Whenever such a predicate fires on a pixel, then a resulting *true* value will be interpreted as 1, which, by way of multiplication, activates the RGB color value associated with it; a value of *false* will make the cell transparent. Some of the optimizations discussed in Section 2.2.4, like just-in-time compilation and GPU exploitation, are especially effective on such queries. The resulting color map is then overlaid with the three dive maps. These already consist of color images, hence no data type adjustment is necessary. Finally, the tile requested is cut out by indicating the bounding box and the CRS in which it is expressed; obviously, first thing an optimizer will do is to first perform the subsetting and then the pixel operations. The final image is encoded in JPEG and delivered to the client.

### 3.3. Three- and Four-Dimensional Data

3-D data often occur as either *x/y/t* time series, or as *x/y/z* earth tomograms, or as *x/y/z* ocean model data. In atmospheric and ocean modeling, 4-D data covering all spatio-temporal dimensions can be found frequently. To conveniently display these in a Web environment, where advanced visualization tools might not fit into the client architecture, they usually are sliced. This yields our next use case:

#### 3.3.1. Scenario 5

**Figure 4.**
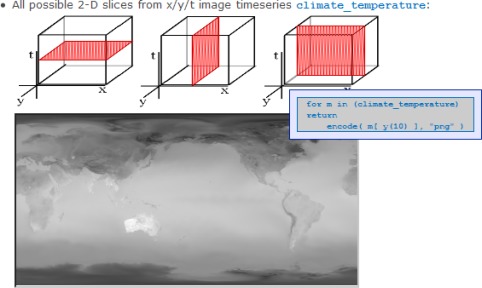
WCPS queries attached to pictograms for slicing of a 3-D cube in EarthLook.

From a 3-D *x/y/t* time series cube, we extract 2-D slices for all three directions (cf. Figure 4). From the three queries we present the one which delivers an *x/z* slice at *x* position *x_0_*.

for $t in (TimeseriesCube)

return

    
encode($t[x:“urn:ogc:def:crs:EPSG::4326”(x_0_)], “jpeg”)

#### 3.3.2. Scenario 6

Here we assume that a satellite image needs to be masked to separate land from sea areas. From some 3-D *x/y/t* time series cube, *Clouds*, the query extracts the slice corresponding to time *T* and overlay it with a 2-D *x/y* Boolean mask, *Land Mask.* This mask is assumed to contain *true* for dry and *false* for wet pixels. For some time *T*, the corresponding WCPS query reads as follows.

**Figure 5.**
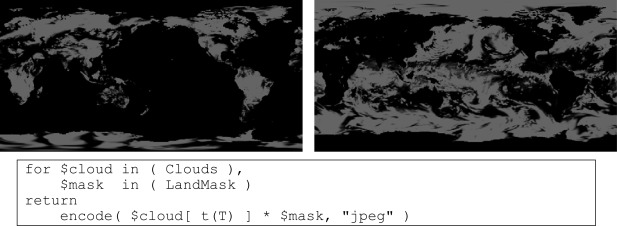
Masking time series images to isolate land (left) and sea areas (right).

Figure 5 shows left the clouds over land. In the right counterpart, $*mask* has been replaced by its negation *not* $*mask*, thereby effectively selecting all clouds over sea.

## 4. Conclusions and Outlook

The abundance of sensor data getting available calls for methods to structure the wealth of information that is becoming accessible. In many important use cases, such as disaster mitigation and scientific serendipity, it is indispensable to enable *ad-hoc* search based on data-inherent criteria. In this respect, WCPS represents an important facet of OGC’s standards for open raster data access. With WCS, a simple service is available which can be implemented with modest effort; WCPS, on the other hand, is a “power service” with high flexibility and expressiveness which comes at an increased implementation effort. Moreover, standardizing a high-level, protocol-agnostic service component facilitates use in different environments. This has been demonstrated already with WCS and WPS; an embedding with SWE seems worthwhile to investigate, as the scenarios presented suggest. This goes hand in hand with the recently started harmonization of SWE with other OGC standards, including WCS.

Considering the sensor data processing chain, WCS and WCPS have their particular place at its end, when consolidated sensor data repositories are made available to either human users or to automated processes. Generally speaking, however, we also see potential application in more upstream tasks where access needs to be based on open standards.

A major innovation in this context is the formal definition of an OGC standard which provides the basis for a machine-readable language. While this may seem more of an academic exercise at first, it has a series of practical consequences: Services can be orchestrated in an unsupervised manner, thereby enabling dynamic, *ad-hoc* mashups. Inside a service node, a host of optimization techniques can be applied which have been proven to boost performance by orders of magnitude [19,20,21]. Altogether, this language concept is considered a substantial step forward towards a Semantic Web which does not only consider metadata, but also sensor data.

After its adoption as a standard in 2008, work on WCPS is ongoing. First and foremost, applications are being sought – which can rely on the readily available open-source service stack – to exercise the raster language in as heterogeneous application scenarios as possible. In particular data centers are encouraged to contact the WCPS team which gladly gives support. To this end, the www.earthlook.org demonstration website will be extended further.

Also, following down the road of SQL, we plan to extend WCPS with manipulation capabilities, similar to the well-known insert, update, and delete statements. This builds a bridge to WCS-T [22] which already allows one to insert, update, and partially update a coverage; the contribution of WCPS can be to allow for complex filter predicates and update processing.

While the flexibility of WCPS is believed an asset it turns out that clients still need to be crafted individually for different communities. To make this work more efficient, a collaboration with GUI specialists has started investigating on a user interface toolkit allowing to quickly pick-and-place widget components while composing tailored WCPS clients. Among others, it will contain controls and display methods for result data of different dimensionality.

Further topics of active research include: theoretical investigations on raster languages and their expressiveness; fully automated decomposition of incoming WCPS requests and their distributed processing in a geo service cloud; extending the coverage model to allow not only regular grids, but general meshes for a WCPS; how to systematically derive quality of service parameters and conformance tests from a specification using state-of-the-art software engineering methods; conversely, how to best structure a specification in a suitable way for conformance testing and orthogonal service unit descriptions. Optimization of processing will continue to be in our focus as the potential is by far not exhausted. In particular, this aspect gets vital when allocating services on board of autonomous units. Goal of this research is to use OGC conformant services as access interfaces to intelligent on-board sensors with low-bandwidth ground connection, such as drones and submersibles.
